# Adiponectinemia Is Associated with Uricemia but Not with Proinflammatory Status in Women with Metabolic Syndrome

**DOI:** 10.1155/2012/418094

**Published:** 2011-07-27

**Authors:** Andréa Name Colado Simão, Marcell Alysson Batisti Lozovoy, Tathiana Name Colado Simão, Helena Kaminami Morimoto, Isaias Dichi

**Affiliations:** ^1^Department of Pathology, Clinical Analysis and Toxicology, University of Londrina, 86038-440 Londrina, PR, Brazil; ^2^Department of Biochemistry and Pathology, University North of Paraná (UNOPAR), Londrina, Paraná, Brazil; ^3^Department of Nutrition, University North of Paraná (UNOPAR), Londrina, Paraná, Brazil; ^4^Department of Internal Medicine, University of Londrina, Londrina, Paraná, Brazil

## Abstract

Metabolic syndrome (MS) is a cluster of glucose intolerance, hypertension, and dyslipidemia with visceral fat accumulation. This study was undertaken to assess which components of metabolic syndrome (MS), including uric acid and proinflammatory markers, are related to adiponectin levels in overweight and obese women with MS. Ninety-one women (60 with MS and 31 controls) were assessed in relation to classical and inflammatory parameters of MS. In comparison to controls, patients with MS showed significant differences in parameters that are typically associated with MS and in inflammatory markers. Fibrinogen, CRP, and C3 were positively, whereas albumin was inversely correlated with abdominal adiposity and insulin resistance. Adiponectin was inversely correlated with waist circumference and uric acid levels. Activities of adiponectin and proinflammatory markers are not correlated in overweight and obese women with MS. In addition to abdominal adiposity, uric acid may be implicated in a decrease of adiponectin in MS patients.

## 1. Introduction

Metabolic syndrome (MS) is a disorder comprised of a combination of glucose intolerance, hypertension, dyslipidemia, and visceral fat accumulation, which promotes the development of cardiovascular diseases and atherosclerosis [1, 2].

Abdominal obesity and insulin resistance are the core features of MS. Inflammation, demonstrated primarily by elevated levels of serum C-reactive protein, is thought to be associated with insulin resistance and MS [3–5]. Central obesity is considered to be one of the most important determinants of the low-grade chronic inflammation present in MS [6].

Adipose tissue produces proinflammatory cytokines, such as interleukin 6 (IL-6), tumor necrosis factor-*α* (TNF-*α*), and complement factors [7]. However, this tissue also secretes adiponectin, a protein showing antiinflammatory activity, which inhibits TNF-*α* production [8], adhesion molecule expression, and nuclear transcriptional factor *κ*B signaling, a pivotal pathway in inflammatory reactions in endothelial cells [9, 10]. In addition, adiponectin is anti-atherogenic and is an insulin-sensitizing agent [11]. Adipose-derived TNF-*α* may have negative effects on the expression of adiponectin and vice versa, and these two proteins also have opposite effects on insulin sensitivity [12, 13]. Given this antagonistic relationship, obesity, and especially visceral obesity, may lead to a decreased secretion of adiponectin through feedback inhibition, thereby suppressing the beneficial effects of adiponectin on insulin sensitivity.

Levels of adiponectin are lower in patients with obesity [14], type 2 diabetes mellitus [15], arterial hypertension [16] and MS [17, 18]. Decreases in serum adiponectin levels are associated with different components of MS, and the decreased adiponectin levels appear to be related to increases in the number of MS components in both sexes [18]. 

In MS pathophysiology, it is unclear whether decreased anti-inflammatory adiponectin and increased proinflammatory markers are associated and occur simultaneously in the development of this syndrome. Some studies have found inverse relationships between adiponectin concentrations and proinflammatory markers [19, 20]. However, a recent study showed that adiponectin levels and proinflammatory status are independent [21]. 

Several studies have demonstrated the importance of uric acid in the physiopathology of MS [22, 23]. In a previous study, we verified a correlation between serum uric acid level and several components of MS, as well as its influence on oxidative stress and antioxidant defense [24]. Nevertheless, few studies to date have assessed the association of uric acid levels with adiponectinemia in MS. 

The knowledge of whether proinflammatory markers and uric acid levels are connected with adiponectin could be important to both the pathophysiology and therapy of MS patients. Therefore, the aim of the present work was to assess which components of MS, including uric acid, and proinflammatory markers, are related to adiponectin levels in overweight and obese women with MS.

## 2. Subjects and Methods

### 2.1. Subjects

Ninety-one women, selected from ambulatory patients and workers of the University Hospital of Londrina, Paraná, Brazil, were chosen to participate in the study. The control group included 31 healthy women, whereas the MS group was made up of 60 overweight and obese women with MS. All women in the control group had a body mass index (BMI) between 20 and 25 kg/m^2^ and did not present any of the metabolic syndrome parameters listed in the definition below. Control group and MS group had 6 and 16 postmenopausal women, respectively. Furthermore, study subjects were not regularly taking any medications. The groups were paired by age, race, smoking habit, and alcohol intake. Information on the lifestyle factors and medical history of the study subjects were obtained through a clinical evaluation. 

MS was defined following the Adult Treatment Panel III criteria [25], when three of the following five characteristics were confirmed: (1) abdominal obesity: waist circumference ≥102 cm in men and ≥88 cm in women; (2) hypertriglyceridemia ≥150 mg/dL (1.695 mmol/L); (3) low HDL cholesterol levels ≤ 40 mg/dL (1.036 mmol/L) in men and ≤50 mg/dL (1.295 mmol/L) in women; (4) high blood pressure (≥130/85 mmHg); (5) high fasting glucose (≥110 mg/dL). 

None of the participants of the study presented thyroid, renal, hepatic, gastrointestinal, or oncological diseases, and none of the participants had a clinically evident infection or were receiving drugs for hyperglycemia, drugs known to affect lipoprotein and uric acid metabolism or inflammatory markers or hormone replacement therapy for at least 4 weeks before the study. All patients provided written informed consent, and the study protocol was fully approved by the Ethical Committee of the University of Londrina (Paraná, Brazil).

### 2.2. Anthropometric and Arterial Pressure Measurements

Height and weight were measured in the morning with subjects wearing light clothing but no shoes. After 5 minutes of rest, each subject had his/her blood pressure measured from the left arm with the subject in a sitting position. We considered the current use of antihypertensive medication to be an indication of high-blood pressure. Body mass index (BMI) was calculated as weight (Kg) divided by height (m) squared. Waist circumference was measured with a soft tape on standing subjects midway between the lowest rib and the iliac crest.

### 2.3. Biochemical Measurements

After fasting for 12 hours, the patients underwent laboratory blood analyses for the following factors: plasma glucose and serum total cholesterol (TC), high-density lipoprotein cholesterol (HDL-cholesterol), low-density lipoprotein cholesterol (LDL-cholesterol), triacylglycerol (TG), and uric acid and albumin levels, which were evaluated by a biochemical auto-analyzer (Dimension Dade AR) using Dade Behring kits. Plasma insulin levels were determined by microparticle enzyme immunoassay (MEIA, AXSYM, ABBOTT Laboratory, Wiesbaden, Germany). All samples were centrifuged at 3.000 rpm for 15 minutes and plasma or serum aliquots were stored at −70°C until they were assayed. Interassay coefficient of variation (CV) for all assays were <10% as determined in human serum.

The homeostasis model assessment (HOMA) was used as a surrogate measure of insulin sensitivity [26] using the following equation: HOMA-IR = insulin fasting (*μ*U/mL) × glucose fasting (nmol/L)/22.5.

### 2.4. Measurement of Inflammatory Markers and Cytokines

The total counts of leucocytes was determined using Cell-Dyn 3700 (ABBOTT Laboratory); plasmatic fibrinogen was measured by the Klauss method; hsCRP (highly sensitive CRP) and serum complement factors C3 and C4 levels were measured using a nephelometric assay (Behring Nephelometer II, Dade Behring, Marburg, Germany). Serum TNF-*α*, IL-6, and adiponectin levels were measured by a sandwich enzyme-linked immunosorbent assay (ELISA) using a commercial immunoassay (R&D System).

### 2.5. Statistical Analysis

Data were expressed as the median values (minimum and maximum). Comparisons between control subjects and patients with MS were conducted using the Mann-Whitney test. Correlations were evaluated by Spearman's rank correlations. The results were considered to be significant when *P* < 0.05. A statistical software program Graph Pad InStat (Graph Pad Software, Inc) was used for analyses.

## 3. Results

Subjects in both groups did not drink alcohol regularly. Clinical and biochemical characteristics of the subjects are shown in Table 1. There were no differences related to age or smoking between the groups. Analyses performed without postmenopausal women did not verify any statistical difference in relation to data shown in the present study. The MS group of patients had significantly higher BMI, waist circumference, systolic and diastolic blood pressure, triacylglycerol levels, fasting glucose and insulin, HOMA, and uric acid levels than the control group (*P* < 0.0001). The MS group also had significantly lower HDL-cholesterol levels (*P* < 0.0001; Table 1).

With regard to the levels of inflammatory markers, patients with metabolic syndrome had significantly higher leukocytes counts (*P* = 0.0004), fibrinogen (*P* = 0.0007), C-reactive protein (*P* < 0.0001), C3 and C4 (*P* < 0.0001), and IL-6 (*P* = 0.0373) concentrations and lower albumin levels (*P* = 0.0146) than the control group. Serum adiponectin levels were significantly lower in the metabolic syndrome group (*P* = 0.0001). TNF-*α* levels were not statistically different (*P* = 0.9952) between the groups (Table 2). 

There were significant correlations between several parameters of metabolic syndrome and the levels of inflammatory markers (Table 3). Waist circumference was positively correlated with leukocyte counts (*r* = 0.25; *P* < 0.05), fibrinogen (*r* = 0.45; *P* < 0.0001), C-reactive protein (*r* = 0.51; *P* < 0.0001), and C3 (*r* = 0.28; *P* < 0.05), and inversely correlated with serum albumin (*r* = −0.37; *P* < 0.01) and adiponectin (*r* = −0.21; *P* < 0.05). 

Diastolic blood pressure was positively correlated with fibrinogen (*r* = 0.30; *P* < 0.05). There was no statistically significant correlation between serum proinflammatory cytokines TNF-*α* and IL-6 and any metabolic syndrome parameter. Serum fasting insulin and insulin resistance assessed by HOMA were positively correlated with fibrinogen (*r* = 0.26; *P* < 0.05), C-reactive protein (*r* = 0.29; *P* < 0.05, and *r* = 0.35; *P* < 0.01, resp.), and C3 (*r* = 0.35; *P* < 0.01, and *r* = 0.42; *P* < 0.001, resp.) and inversely correlated with serum albumin (*r* = −0.30; *P* < 0.05, and *r* = −0.29; *P* < 0.05, resp.). Serum triacylglycerol levels were positively correlated with C3 (*r* = 0.34; *P* < 0.01), and C4 (*r* = 0.42; *P* < 0.001; Table 3). 

**Figure 1:**
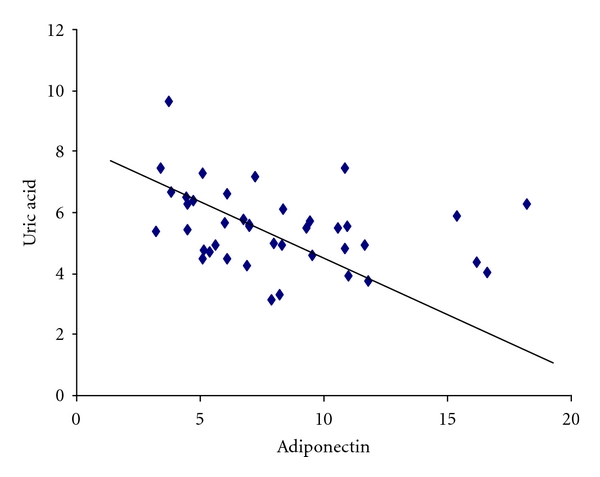
Dispersion graphic between plasma levels of uric acid and adiponectin in patients with metabolic syndrome (*r* = −0.3752; *P* = 0.0170).

Serum adiponectin levels were not correlated with any proinflammatory markers in the present study (Table 4). However, serum uric acid levels were inversely correlated with adiponectin (*r* = −0.3752; *P* < 0.0170; Figure 1).

## 4. Discussion

The major findings of the present study were that adiponectin was inversely associated with uric acid levels and that adiponectin and the activity of proinflammatory markers are not correlated in patients with MS. The present study also evaluated several inflammatory markers in subjects with MS. The results are in agreement with the literature, showing that patients with MS present a higher proinflammatory state and a decrease in anti-inflammatory mediators, shown by reduced levels of adiponectin [4, 27].

The present work also verified that aside from C4, TNF-*α*, and IL-6, inflammatory markers were associated with waist circumference and insulin resistance, emphasizing that this syndrome is proinflammatory. Therefore, aside from CRP, both positive (fibrinogen and C3) and negative acute phase reactant proteins (albumin) can be considered inflammatory markers of good accuracy in MS. 

 Abdominal subcutaneous tissue produces a variety of adipokines, such as TNF-*α* and IL-6, which has an important role in inflammation and insulin resistance via endocrine, paracrine, or autocrine signals [28–30]. Interleukin-6 (IL-6) is considered to be the major mediator of the hepatic acute-phase reaction and is thought to play a central role in the pathogenesis of cardiovascular disease in patients with insulin resistance [31]. The data in the present study are consistent with those in the literature which show that CRP is more closely related to glucose metabolism alteration than IL-6, since CRP has a significantly increased half life in relation to IL-6 [32]. 

Although serum IL-6 levels were significantly higher in the patients with MS than in controls in the current study, levels of TNF-*α* were not found to be higher in MS patients. TNF-*α* does not seem to be released into the circulation and is, thus, unable to signal systematically [33, 34], thus, functioning as a paracrine pathway [12]. In accordance with a previous study [27], our data also showed that the level of serum TNF-*α* is not an appropriate inflammatory marker in patients with MS. 

Adipose tissue is a direct source of complement factors. Several studies have shown that complement factors strongly predict cardiovascular events in adolescents and adults [35, 36]. Central obesity in particular seems to trigger C3 production [36]. The results of the current study confirmed the findings of the correlation between central obesity and C3. Moreover, C3 is also correlated with insulin resistance and triacylglycerol.

The results of the present study are in agreement with the findings of a correlation between central obesity and CRP. In addition, CRP was also correlated with fasting insulin and insulin resistance verified by HOMA. Similarly to the results of the current study, meta-analysis of prospective studies showed a positive correlation between coronary heart disease and fibrinogen, C-reactive protein, and leukocyte counts, and a negative correlation with albumin [37]. Therefore, in the present study, inflammatory markers were more closely related to abdominal adiposity and insulin resistance than to dyslipidemia and arterial hypertension. 

As reported in other studies, lower levels of adiponectin were observed in MS patients. Furthermore, adiponectin was correlated with WC in MS patients but was not related to any proinflammatory marker in either MS patients or in the control group (data not shown). Yang et al. [38] also showed that in subjects with BMI > 35 kg/m^2^, adiponectin levels were not significantly related to most variables of MS, except for the waist-to-hip ratio. They suggest that this finding may simply imply that “body-weight factors” could be more important than other factors in modulating plasma adiponectin levels in their study. Matsushita et al. [27] showed a stronger association between adiponectin and parameters of MS than with IL-6, TNF-*α*, and even CRP. The authors concluded that adiponectin plays a key role in the development of MS and that determining serum adiponectin levels is important for the prevention and treatment of this syndrome. 

Herder et al. [21] have not found a correlation between serum adiponectin and several proinflammatory markers in patients with an impaired glucose tolerance and type 2 diabetes mellitus. The authors suggested that hypoadiponectinemia and low-grade inflammation are independent and distinct factors. 

In the meantime, a growing body of evidence indicates that elevated uric acid levels are commonly associated with cardiovascular disease, and uric acid has even been suggested to have a causal role in hypertension and MS [23]. Uric acid traverses dysfunctional endothelial cells and accumulates as crystals within atherosclerotic plaques. These crystals may contribute to local inflammation and plaque progression [39]. Uric acid has been shown to activate leukocytes and stimulates the production of interleukin 1-*β*, interleukin 6 (IL-6), and TNF-*α* by mononuclear cells, and CRP by cultured human vascular cells [22, 39]. Nevertheless, our data have not shown any significant association between uric acid levels and inflammatory markers, except for adiponectin.

Patel et al. [40] also verified that adiponectin was inversely associated with uric acid in apparently healthy young adults. The authors concluded that the functional metabolic role of adiponectin in this inverse relationship, independent of the other known biologic factors such as insulin resistance and visceral adiposity, was not clear. Adiponectin was the unique inflammatory marker measured in the current study that was correlated with serum uric acid levels (an inverse correlation), and both were significantly altered in MS patients. On the other hand, adiponectin levels were not correlated with uric acid levels in the control group (data not shown).

Although uric acid may have a protective effect due to its antioxidant properties [38], it is clear that the dominant effect of uric acid in MS is deleterious. It is still unclear which of the following detrimental roles of uric acid is more important: mediating the effects of conventional risk factors in the development of the atherosclerotic disease, mediating the effects of an anti-inflammatory status, or mediating the effects of a proinflammatory status. Our data, which showed an inverse relationship between adiponectin with WC and uric acid levels, reinforce the former two hypotheses. 

When considering the results of the present study, the following limitations must be considered: first, the small size sample did not allow for a correction for multiple testing. Second, the present study was performed with pre- and postmenopausal women what could have interfered with the results, although statistical analyses have not confirmed this likelihood. Third, some of the variables may have been confounding. Therefore, the data shown in the present study should be considered carefully. Nevertheless, we conducted rigorous clinical and laboratorial assessments to ensure that the control and patient groups did not present any conditions which could interfere with the research, such as chronic diseases and drugs which could influence inflammatory markers, uric acid levels, or metabolic syndrome parameters. Thus, we were confident that the patients in this study had a unique diagnosis of MS. Furthermore, subjects in this study were exclusively women, which were paired by age, race, smoking habit, and alcohol intake.

## 5. Conclusion

In conclusion, patients with MS presented several increased inflammatory markers that were primarily associated with insulin resistance and abdominal adiposity. The decrease in anti-inflammatory protection and the increase in the inflammatory process seem to be independent processes in MS patients. Except for an inverse correlation with adiponectinemia, serum uric acid levels did not correlate with any other inflammatory marker. Although a transversal study does not necessarily imply causality, it does suggest a role of uric acid in the etiology of hypoadiponectinemia in MS. Further investigations are needed to confirm this likelihood.

## Figures and Tables

**Table 1:** Clinical and laboratory characteristics of patients with metabolic syndrome and controls.

	Metabolic Syndrome (*n* = 60)	Controls (*n* = 31)	*P*
Age (years)	45.0 (25.0–60.0)	41.5 (25.0–54.0)	0.1257
Smoking/no smoking	2/58	1/30	0.6379
BMI (m/kg^2^)	37.2 (27.1–53.9)	22.9 (20.1–24,5)	<0.0001
WC (cm)	110.0 (88.0–157.0)	79.0 (61.0–86.0)	<0.0001
SBP (mmHg)	134.5 (99.0–203.0)	101.0 (81.0–130.0)	<0.0001
DBP (mmHg)	83.0 (62.0–124.0)	65.0 (47.0–80.0)	<0.0001
Triacylg (mg/dL)	202.0 (40.0–401.0)	70.0 (32.0–135.0)	<0.0001
HDL (mg/dL)	39.0 (19.0–81.0)	58.5 (51.0–77.0)	<0.0001
Fasting glucose (mg/dL)	100.5 (74.0–195.0)	80.0 (71.0–93.0)	<0.0001
Fasting insulin (*μ*U/mL)	18.50 (4.10–75.40)	4.40 (2.90–9.80)	<0.0001
HOMA	4.47 (0.83–36.24)	0.84 (0.57–1.79)	<0.0001
Uric acid (mg/dL)	5.51 (3.15–9.65)	3.38 (2.07–4.73)	<0.0001

Data are median (minimum–maximum).

BMI: body mass index; WC: waist circumference; SBP: systolic blood pressure; DBP: diastolic blood pressure; Triacylg: triacylglycerol; HDL: High-density lipoprotein; HOMA: Homeostasis model of assessment.

**Table 2:** Cytokines, inflammatory markers and adiponectin levels in patients with metabolic syndrome and controls.

	Metabolic Syndrome (*n* = 60)	Controls (*n* = 31)	*P*
Leukocytes (*μ*L^−1^)	7700 (3500–13800)	6000 (3100–8600)	0.0004
Fibrinogen (mg/dL)	314.0 (185.0–489.0)	262.0 (188.0–314.0)	0.0007
CRP (mg/L)	7.80 (1.10–46.5)	0.70 (0.16–5.20)	<0.0001
Albumin (g/dL)	4.41 (3.56–5.55)	4.74 (3.75–5.47)	0.0146
C3 (mg/dL)	202.8 (115.0–271.0)	126.0 (94.8–188.0)	<0.0001
C4 (mg/dL)	43.20 (24.00–96.60)	23.50 (14.60–43.10)	<0.0001
TNF-*α* (pg/mL)	32.69 (11.62–193.81)	38.71 (8.58–86.26)	0.9952
IL-6 (pg/mL)	3.96 (3.04–92.45)	3.32 (2.55–9.63)	0.0373
Adiponectin (*μ*g/mL)	7.11 (3.19–18.22)	12.31 (9.11–27.27)	0.0001

Data are median (minimum-maximum).

CRP: C-reactive protein; C3: complement factor C3; C4: complement factor C4; TNF-*α*: tumor necrosis factor-*α*; IL-6: interleukin-6.

*P ≤ 0.05, ^¤^ *P* ≤ 0.01, ^‡^ *P* ≤ 0.0001.

**Table 3:** Spearman's Correlation coefficients among inflammatory markers and the components of metabolic syndrome.

	Leuk	Fib	CRP	C3	C4	Alb	TNF-*α*	IL-6	ADIP
WC	0.25*	0.45^‡^	0.51^‡^	0.28*	−0.07	−0.37^¤^	−0.15	0.07	−0.21*
SBP	0.18	0.15	0.14	0.10	−0.05	−0.06	0.07	−0.06	0.09
DBP	0.21	0.30*	0.19	0.22	0.02	−0.01	0.01	0.17	−0.12
Triacylg	0.11	−0.22	0.04	0.34^¤^	0.42^†^	0.11	0.10	0.10	−0.05
HDL	0.12	0.05	−0.02	−0.05	−0.10	0.01	0.05	0.06	0.15
Glucose	0.17	0.12	0.12	0.20	−0.01	−0.12	−0.04	−0.10	−0.10
Insulin	0.10	0.26*	0.29*	0.35^¤^	−0.10	−0.30*	0.04	0.13	−0.18
HOMA	0.21	0.26*	0.35^¤^	0.42^†^	−0.07	−0.29*	−0.04	0.13	−0.18

Leuk: leukocytes; Fib: fibrinogen; CRP: C-reactive protein; C3: Complement factor C3; C4: Complement factor C4; Alb: albumin; TNF-*α*: tumor necrosis factor-*α*; IL-6: interleukin-6; Adip: adiponectin; WC: waist circumference; SBP: systolic blood pressure; DBP: diastolic blood pressure; Triacylg: triacylglycerol; HDL: High-density lipoprotein; HOMA: Homeostasis Model of Assessment.

*P ≤ 0.05, ^¤^ *P* ≤ 0.01, ^†^ *P* ≤ 0.001, ^‡^ *P* ≤ 0.0001.

**Table 4:** Spearman's Correlation coefficients among adiponectin and the inflammatory markers in patients with metabolic syndrome.

	Adiponectin (*n* = 60)	
	*R*	*P*
Leukocytes	−0.291	.065
CRP	−0.247	.119
Fibrinogen	−0.126	.427
Albumin	−0.065	.991
C3	−0.24	.134
C4	−0.12	.452
TNF-*α*	−0.156	.330
IL-6	−0.002	.991

CRP: C-reactive protein; C3: complement factor C3; C4: complement factor C4; TNF-*α*: tumor necrosis factor-*α*; IL-6: interleukin-6.
